# Toward Advanced Functional Systems: Honeycomb-Like Polymeric Surfaces Incorporating Polyoxovanadates with Surface-Appended Copper-Cyclam Complexes

**DOI:** 10.3390/molecules24122313

**Published:** 2019-06-22

**Authors:** Leire Ruiz-Rubio, Beñat Artetxe, Leyre Pérez-Álvarez, Jagoba Martín-Caballero, Tatsumi Ishihara, Juan M. Gutiérrez-Zorrilla, José Luis Vilas-Vilela

**Affiliations:** 1 Grupo de Química Macromolecular (LABQUIMAC). Departamento de Química Física, Facultad de Ciencia y Tecnología, Universidad del País Vasco UPV/EHU, P.O. Box 644, 48080 Bilbao, Spain; leyre.perez@ehu.eus; 2 BCMaterials, Basque Center for Materials, Applications and Nanostructures, UPV/EHU Science Park, 48940 Leioa, Spain; jagokun19@gmail.com (J.M.-C.); juanma.zorrilla@ehu.eus (J.M.G.-Z.); 3 Departamento de Química Inorgánica, Facultad de Ciencia y Tecnología, Universidad del País Vasco UPV/EHU, P.O. Box 644, 48080 Bilbao, Spain; benat.artetxe@ehu.eus; 4 International Institute for Carbon-Neutral Energy Research (I2CNER), Kyushu University, Fukuoka 819-0395, Japan; ishihara@cstf.kyushu-u.ac.jp

**Keywords:** polyoxometalates, breath figures, surface functionalization

## Abstract

In this work the immobilization of hybrid polyoxometalates (POMs) onto functional polymeric surfaces is exposed and discussed. Thus, various hybrid polymer‒inorganic films were prepared by anchoring selected hybrid POMs onto tailored polymeric surfaces that consisted of breath figures (BFs) made of polystyrene-b-poly(acrylic acid)/polystyrene (PS-b-PAA/PS) blends. Functionalization of the BF films was performed by selective arrangement of acrylic acid groups of the amphiphilic block copolymer on the surface pores because of their affinition for the water condensed during breath figure formation. These carboxylic acid functional groups contained within the PAA blocks were then employed to anchor [Cu(cyclam)][{Cu(cyclam)}_2_(V_10_O_28_)]·10H2O (**1-CuV10**) and [{Cu(cyclam)}(VO_3_)_2_]·5H_2_O (**1-CuV1**), hybrid POMs by immersing the films into aqueous solutions of the in situ formed hybrid clusters, resulting in the hybrid films **BF1** and **BF2**, respectively. Superficial analysis of these hybrid polymeric films was carried out by the sophisticated ion beam-based technique time-of-flight secondary ion mass spectrometry (ToF-SIMS) that was revealed to be an excellent method for the superficial compositional mapping of patterned surfaces.

## 1. Introduction

Preparation of functional polyoxometalate (POM)-based materials is one of the hot topics within the chemistry of this family of nanometric metal-oxo clusters. They have outstanding properties and are applicable in current issues of interest related to technology, health, energy, and the environment [1,2,3]. Despite their potential, direct preparation of such materials has been rather scarce because POMs usually present as crystalline solids that are hard to process. By reducing their structural dimensions from bulk powders to low‒dimensional structures (i.e., bearing at the surface of nanoparticles or interphases of films), these nanoclusters can be used as components for functional devices such as electrodes, electronic circuits, heterogeneous catalysts, and sensing materials, among others [4,5,6].

Recently, POMs have been incorporated into different types of materials, such as carbon nanotubes, metallic nanoparticles, polymeric matrixes, or even proteins by different methods like physical blending, electrostatic interactions, supramolecular modifications of the clusters (e.g., micelles), or covalent bonding [7,8,9,10]. When it comes to polymeric materials, it has been shown that they can be covalently included in either polymer backbones or pending side chains and also act as crosslinking units [11,12]. However, immobilization of POMs onto solid surfaces still represents a key step toward processing these nanoclusters into practical devices [13]. For instance, heterogenization of a given catalytically active species could considerably increase its surface area and respond to a clear demand from industry in terms of recyclability, which allows the same catalyst to be reused several times [14,15]. Different solid substrates have been used as supports, such as oxides (alumina, silica), metals (silicon and gold), organic polymers, or graphene [16,17,18,19,20,21,22]. Although ionic interactions have been identified as the simplest way to immobilize polyanions [23], the electrostatic forces between POMs and positively charged regions of the surface might lead to partial leaching of these clusters. In order to overcome these difficulties, two main covalent strategies have been used to link POMs to solid surfaces [24,25,26,27]. The first method consists of direct reactions between organically derivatized POMs and substrates bearing complementary functionalities (e.g., amino and carboxylic groups as well as azide and alkine groups via click chemistry) [28,29,30,31]. Nevertheless, highly elaborate functionalities for applications in, for example, optics, molecular electronics, or sensing often require postfunctionalization of preformed hybrid POM platforms via multistep synthetic work [32,33,34]. Some other authors have opted for combining metal-substituted POMs that have exposed and accessible centers with solid substrates bearing functional groups that have coordinating ability [35,36]. Following the coordinative strategy, some of us reported [37] a very facile approach to incorporate lanthanide-containing Keggin-type [Ln^III^(SiW_11_O_39_)]^5−^ (Ln = Ce, Er) species on spin-coated polystyrene-b-poly(acrylic acid)/polystyrene (PS-b-PAA/PS) films. Polymeric surfaces were functionalized through interfacial migration of carboxylate groups upon water annealing. These organic functions acted as anchoring points for metal-substituted POMs with available coordination positions via chelation.

Regarding advanced polymeric films, the breath figure (BF) process is a cost-effective method for preparing highly ordered, honeycomb-patterned films with high surface area from polymer solutions. In this process, a polymer solution is casted onto a substrate under adequate humidity, and evaporation of the solvent cools down the solvent/air interface to induce the condensation of small water droplets [38]. Variations of the concentration, solvent, and relative humidity enables control over the formation of honeycomb patterns [39]. Their fabrication has received an increasing interest because of the potential applications of these structures in different areas such as tissue engineering, membranes, or catalysis [40,41]. To our knowledge, there are only few examples of such polystyrene-based, honeycomb-like polymeric surfaces decorated with POM anions, but all of them involve the use of surfactant-encapsulated polyanions in their fabrication process. It is worth highlighting the luminescent properties of some of these systems. Their uses in biodetection and selective adsorption of proteins, as well as their potential application in catalysis, sensing, or separation membranes, are mentioned by other authors [42,43,44,45]. 

Herein we present a postsynthetic method for the immobilization of POM units based on a similar coordinative approach mentioned above, but instead we use a transition metal belonging to a metalorganic complex covalently linked at the POM surface as an anchorage point. For this purpose, we focused our studies on polyoxovanadate species appended with copper(II) complexes of N-donor ligands as simple models of hybrid POM–metalorganic systems. Plasticity of the coordination sphere of the Cu^II^ centers and the axial elongation they undergo when involved in octahedral environments should enhance the role of the complexes as flexible linkage positions. From the extensive work carried out to date on hybrid vanadate/{Cu^II^(organoamine)}-complex systems [46,47,48], we selected two open-framework materials formed by the assembly of polyoxovanadates and surface-appended {Cu(cyclam)}^2+^ complexes (cyclam = 1,4,8,11-tetraazacyclotetradecane), namely [Cu(cyclam)][{Cu(cyclam)}_2_(V_10_O_28_)]·10H_2_O (**1-CuV10**) and [{Cu(cyclam)}(VO_3_)_2_]·5H_2_O (**1-CuV1**) [49,50]. It is worth highlighting that the former hybrid displays remarkable activity as a heterogeneous catalyst toward H_2_O_2_-based oxidation of the highly stable, tricyclic alkane adamantane. To immobilize these hybrid POM species, honeycomb patterned surfaces were prepared by static breath figure methods from PS-b-PAA/PS blends because this technique allowed the selective functionalization of the formed patterns as the acrylic acid blocks were exclusively located in the cavities. Thus, carboxylic acid functional groups contained within the PAA blocks were used to coordinate **1-CuV10** and **1-CuV1** POM–metalorganic systems by immersing the films into aqueous solutions of the in situ formed hybrids. Functional films were characterized by time-of-flight secondary ion mass spectrometry (ToF-SIMS), which is a powerful technique because of its analytical capabilities for the determination of chemical and isotopic compositions at surface and near-surface regions [51,52]. In comparison to other superficial characterization techniques, such as X-ray photoelectron spectroscopy (XPS), it allows compositional mapping of patterned surfaces to unequivocally determine the exact location of inorganic entities in our hybrid systems.

## 2. Results and Discussion

### 2.1. Preparation of the Breath Figure (BF) Surface Samples

**Figure 1:**
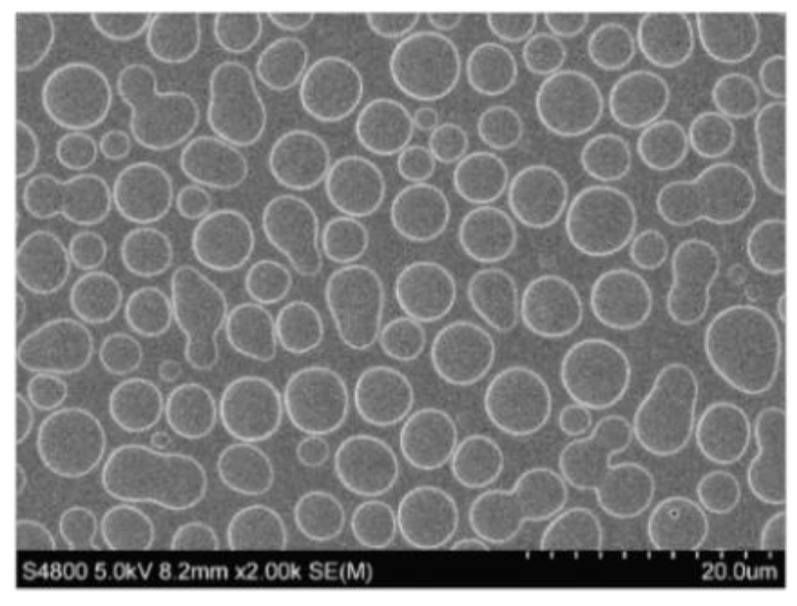
SEM image of the honeycomb-like arrays of polystyrene-b-poly(acrylic acid)/polystyrene (PS-b-PAA/PS) blends in tetrahydrofuran (THF) obtained by the breath figure (BF) method.

The static breath figure method [53] was used to deposit a polymeric film on a glass substrate with micrometric cavities. An SEM image of an example of the honeycomb patterns obtained by the static breath figure methods at 80% relative humidity (RH) from the solution of PS/PS-b-PAA blends is shown in Figure 1. The hexagonal array of microcavities in the polymer film seemed to be polydisperse and slightly organized, as it was usually observed when using this specific fabrication method. Honeycomb-like patterned films decorated with poly(acrylic acid) blocks in the holes were immersed in aqueous hybrid POM solutions. Thus, inorganic entities were immobilized by their interaction with poly(acrylic acid) branches located primarily at the cavities as a result of the affinity of the groups for the droplets involved in breath figure formation [54]. In addition, considering the pH of aqueous POM solutions in the preparation of hybrid surfaces **BF1** (pH ≈ 5) and **BF2** (pH ≈ 9), it is worth noting that the majority of the carboxylic groups should be in a deprotonated state (pKa ≈ 4.5) [55]. Thus, the electrostatic repulsion between functional groups i) minimized the possibility of forming hydrogen bonds between POMs and the PAA, and ii) the swelled topography within the cavities, reported in similar related studies [56], maximized the chances for their coordination to the accessible positions in the POM-appended Cu(cyclam) complexes.

**Figure 2:**
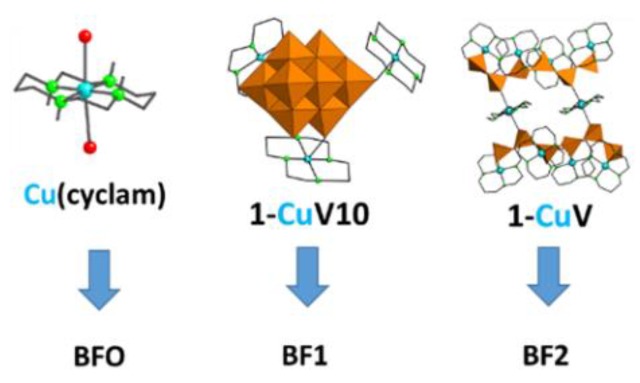
Structures of the inorganic systems anchored to acrylic acid groups presents in the breath figure surfaces.

Hybrid POMs containing copper(II) complexes of the macrocyclic cyclam ligand were selected because of the plasticity of the coordination sphere of the Cu^II^ centers. It is important to notice that the cyclam ligand allows axial positions of the coordination sphere of the metal to be available for coordinating these carboxylic functions present in block copolymers mainly located on the cavities of the films. As described in the experimental section, honeycomb-patterned surfaces were immersed in solutions of the in situ generated **1-CuV10** and **1-CuV** POM hybrids, which resulted in the functional surfaces **BF1** and **BF2**, respectively. For comparison, a film with [Cu(cyclam)(H_2_O)_2_]SO_4_ (hereafter, Cu(cyclam))complex) anchored on the surface (BF0) was used as reference (Figure 2). In order to analyze the sample composition both at the outermost area of the film and a depth profile, samples were characterized by ToF-SIMS. In addition, optical images were obtained with a 3D laser microscope to analyze the roughness of the samples.

### 2.2. Spectroscopic Characterization of BF Samples

**Figure A1:**
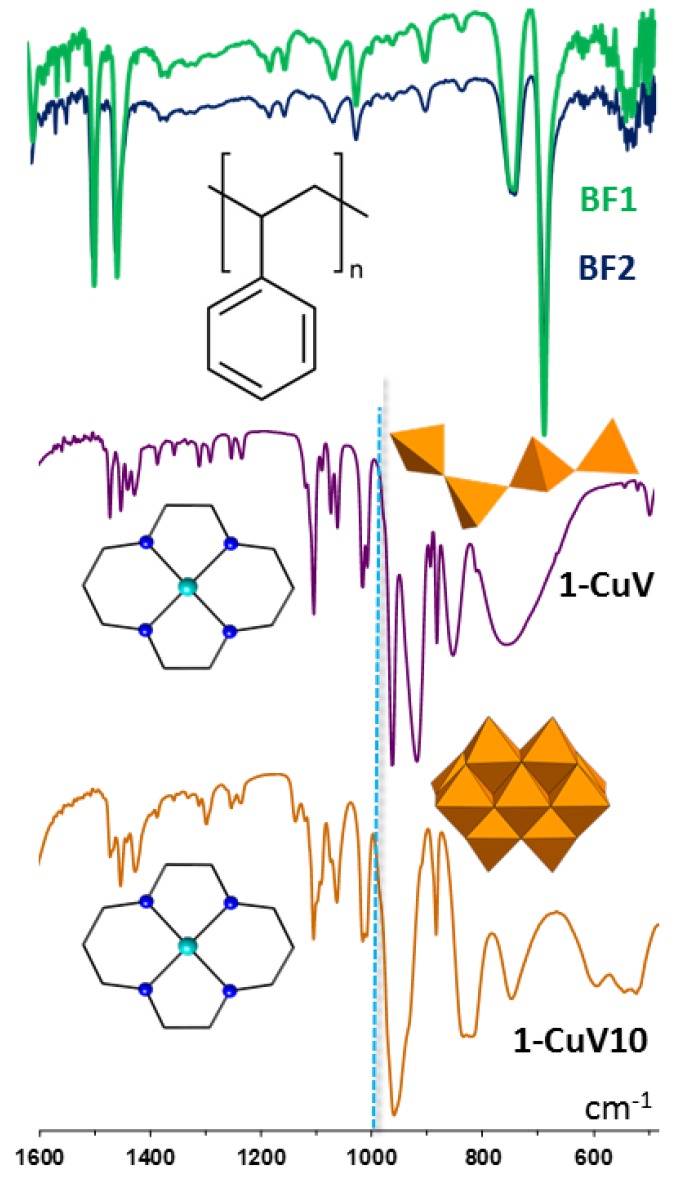
ATR-FTIR spectra of **BF1**, **BF2,** and solid samples of **1-CuV** and **1-CuV10**.

To confirm the presence of POM species in polymeric surfaces and determine whether the hybrid species remained intact or decomposed during the process, we performed attenuated total reflectance Fourier-transform infrared (ATR-FTIR) spectroscopy and diffuse reflectance (DR)-UV/Vis experiments. First, we selected ATR-FTIR spectroscopy to analyze the functional **BF1** and **BF2** surfaces and compared their spectra with those registered for solid samples of **1-CuV** and **1-CuV10.** The spectra of **1-CuV** and **1-CuV10** in the 500–1600 cm^−1^ range displayed two well-differentiated parts: the region below 1000 cm^−1^ showed characteristic bands of corresponding polyoxovanadate units (metavanadate and decavanadate species, respectively), whereas vibrational bands belonging to {Cu(cyclam)}^2+^ species were observed above 1000 cm^−1^ [49,50]. For comparison, the spectra from the hybrid surfaces **BF1** and **BF2** were identical to each other and did not exhibit any of these bands (Figure A1). Furthermore, they were very similar to those expected for a pure polystyrene matrix [57], which was the main component of the block copolymers used for the preparation of breath figure surfaces. Therefore, we concluded that the low concentration of hybrid POMs in the surface prevented us from identifying their presence by ATR-FTIR.

**Figure 3:**
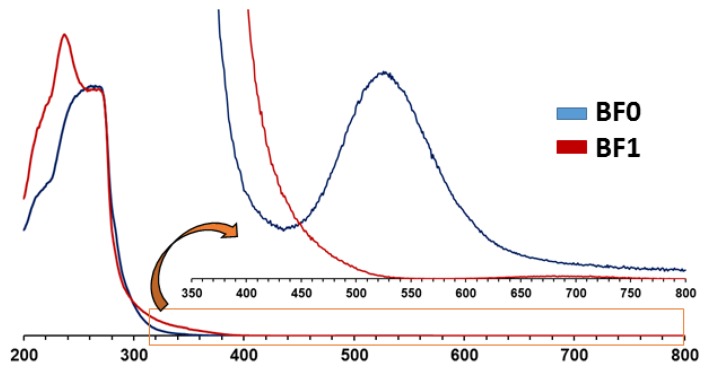
Diffuse reflectance UV/Vis spectra of **BF0** and **BF1** hybrid surfaces and expanded 350–800 nm region.

The pale color of the hybrid surfaces was similar to their parent solutions, which suggested that POM–metalorganic systems remained intact upon immobilization. Thus, taking the **BF1** system as a representative example, we decided to perform diffuse reflectance UV/Vis analyses on **BF0** and the cited **BF1** samples. Both spectra recorded from 200 to 800 nm are depicted in Figure 3 and exhibit a broad band spanning from about 200 to 400 nm. In the case of **BF0**, this band was composed of two maxima centered at about 230 and 280 nm, which might be attributed to π−π* transitions of the aromatic groups belonging the polystyrene, whereas that of BF1 displayed an additional band centered at 250 nm, which could be associated with the ligand to metal (V→O) charge transfer. [58] This observation confirmed the presence of the decavanadate anion in the **BF1** sample. Close inspection to the expanded 450–800 nm region of the spectra revealed a third band centered at 525 nm (green) for {Cu(cyclam)}^2+^ complexes in **BF0**, whereas no additional absorption was observed for **BF1**. This fact indicated that no free {Cu(cyclam)}^2+^ complex was found in the latter surface, and, therefore, no phase segregation took place throughout its preparation process. The green absorption was on the origin of the pink color of **BF0**, whereas the longer tail of the strong band mentioned before in the UV region for **BF1** resulted in a strong absorption in the purple region; therefore, the surface acquired a pale orange-brown color.

### 2.3. Superficial Characterization of BF Samples

**Figure 4:**
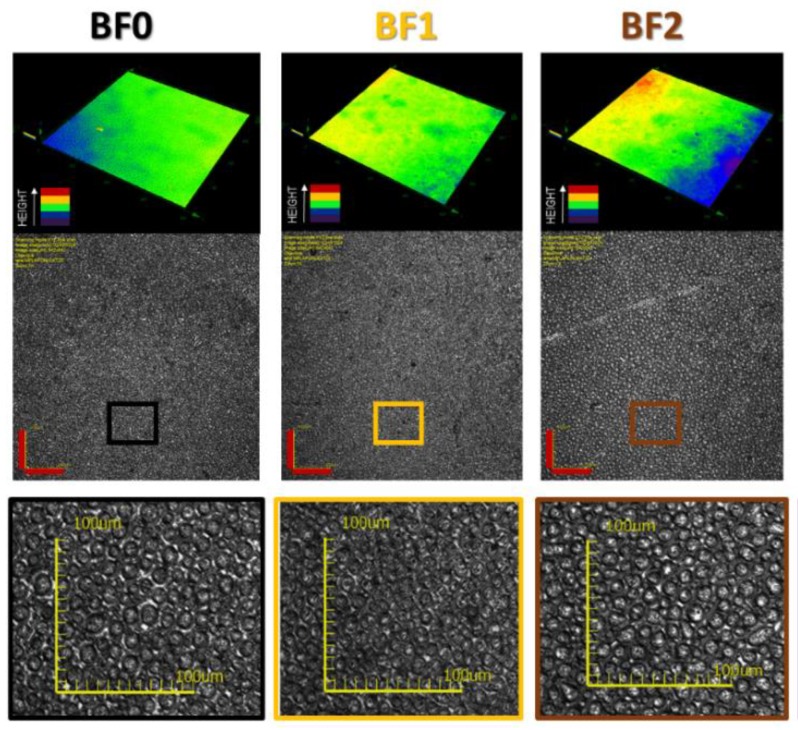
Optical microscope images of the **BF0**, **BF1,** and **BF2** samples: height color map and magnification of the selected area (colored square) (scale bar = 100 μm).

First, the topography of the samples and their roughness were analyzed by optical microscopy (Figure 4). Samples presented a good distribution of cavities in the surface that could be adequate for future applications, so the obtained honeycomb patterns could be suitable for anchoring polyoxometalate anions. In addition, the roughness of the surfaces could prevent adequate measurement of the surface composition by ToF-SIMS, as it was important to corroborate that the samples to be analyzed presented low roughness. The color maps in Figure 3 indicate the height of the analyzed surface. The blue and green color represent the optimum height to be measured by this technique, and the red is the limit for it. As it could be observed, the sample **BF2** presented a slightly rougher surface than the rest of the samples; however, this was still appropriate for the ToF-SIMS analysis.

**Figure 5:**
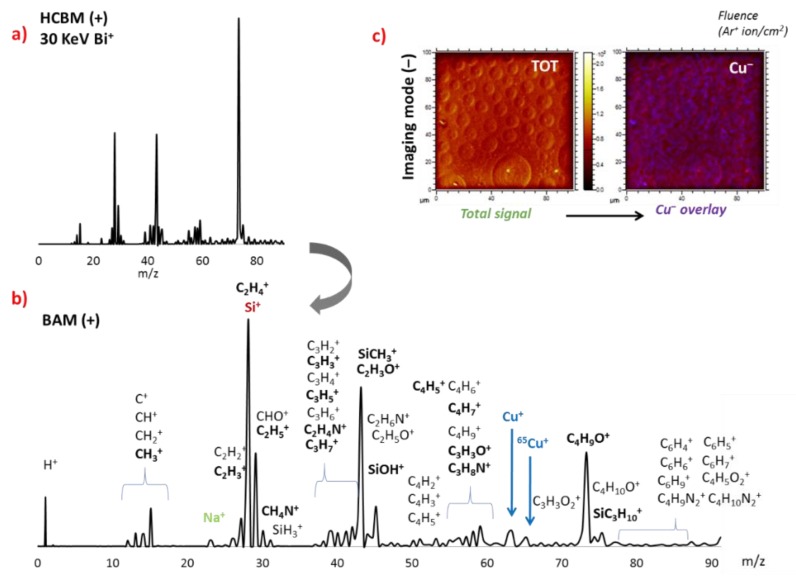
(**a**) 30 KeV Bi^+^ secondary ion mass spectrometry (SIMS) (+) spectrum of BF0 recorded in high-current bunch mode (HCBM). (**b**) Burst alignment mode (BAM) with assignment of the most probable fragments of the organic components and metals, highlighting the strongest. (**c**) Surface images obtained in imaging mode (‒) are also shown and indicate the spatial distribution of the signals arising from the Cu^−^ peaks.

Confirmation of the immobilization of POM on the film surfaces was carried out by ToF-SIMS experiments. First, the **BF0** surface containing exclusively the Cu(cyclam) complex was measured as a reference sample. The mass spectrum working in positive mode revealed the most probable fragmentation modes of the organic components spanning in the *m/z* range from 10 to 90. These signals could be assigned to aliphatic groups of different lengths, C1–C6 (Figure 5a,c), and they evidenced the presence of cyclam ligands at the outer layers of the samples. Similarly, the presence of copper was confirmed by the characteristic peaks of Cu^+^ (*m/z* = 63) and its isotope ^65^Cu (*m/z* = 65). Strong signals corresponding to Si^+^ (*m/z* = 28) and related peaks (SiH_3_^+^, SiOH^+^) were also found, which could be easily associated with the glass support and the preparation method because they were observed in all BF samples. In close analogy, a small peak corresponding to Na^+^ (*m/z* = 23) could also be identified, which is a common residue that is often detected when measuring in positive mode [59]. As it could be observed in Figure 5c, the SIMS images obtained in negative mode showed the pores of the surface that did not suffer notable changes compared to the blank surfaces observed in SEM prior to immobilization of the POMs. Interestingly, the overlay of the Cu^−^ signal versus the total signal suggested that the positions where the Cu signal originated were mainly located at the pores. That is, the metalorganic complex primarily interacted with acrylic acid groups present in the cavities.

**Figure 6:**
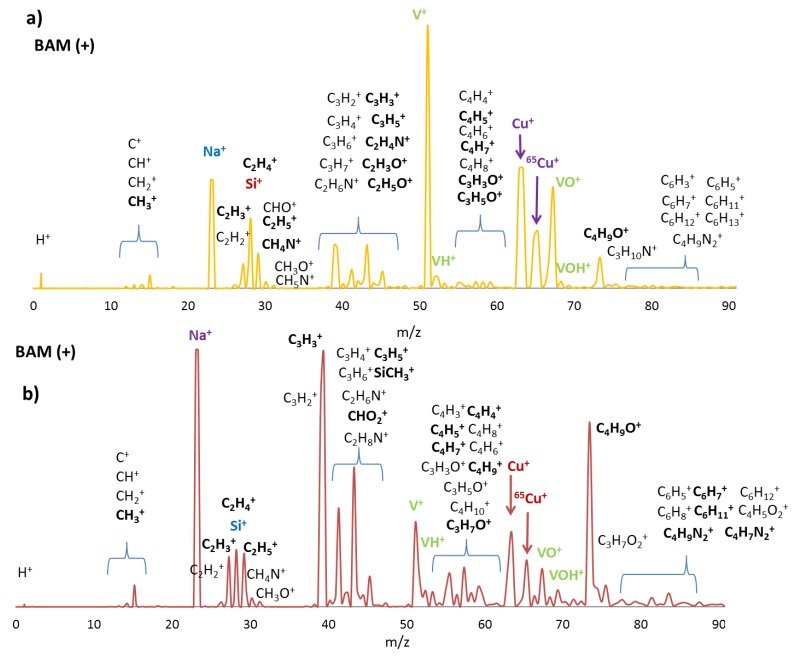
30 KeV Bi^+^ SIMS (+) spectrum recorded in BAM with assignment of the most probable fragments of the organic components and metals, highlighting the strongest signals for (**a**) **BF1** and (**b**) **BF2**.

Some notable changes in the SIMS (+) spectra of both **BF1** and **BF2** anchored on the surfaces were observed compared to the reference BF0 (Figure 6a and Figure 6b, respectively). The presence of high intense peaks associated to V, such as V^+^ (*m/z* = 51) and VO^+^ (*m/z* = 67), added to less intense VH^+^ and VOH^+^ peaks. Figure 6a indicated that {V_10_O_28_} species were located at superficial layers of the film and confirmed successful anchoring of **1-CuV10** to the PAA-functionalized cavities. In addition, the intensity of the Na^+^ (*m/z* = 23) peak increased when compared to the reference (as it could be expected) since **1-CuV10** was synthetized in NaCl 1 M medium, and some cationic moieties could remain in the polymeric surface. Similar to that observed for **BF0**, signals corresponding to Cu^+^ and ^65^Cu^+^ were found in the mass spectrum. In contrast, the Si^+^ and related peaks decreased significantly. 

The positive SIMS (+) spectrum obtained from **BF2** sample presented some similarities with that of **BF1.** The characteristic peaks of V^+^ (*m/z* = 51), Cu^+^ (*m/z* = 63), and organic fragments were observed in Figure 6b and confirmed the presence of the hybrid POM. In addition, a decrease in V^+^ and related peaks could be observed with respect to **BF1,** which could be related to the chemical structure of both hybrids. The atomic Cu/V ratio for the **1-CuV10** hybrid contained in **BF1** was 3/10, whereas that in **1-CuV** present in **BF2** was 1/2. This was in good agreement with the relative intensities of the Cu^+^/V^+^ signals observed in both SIMS spectra, considering there was an excess of free Cu(cyclam) used in the synthetic procedure.

**Figure 7:**
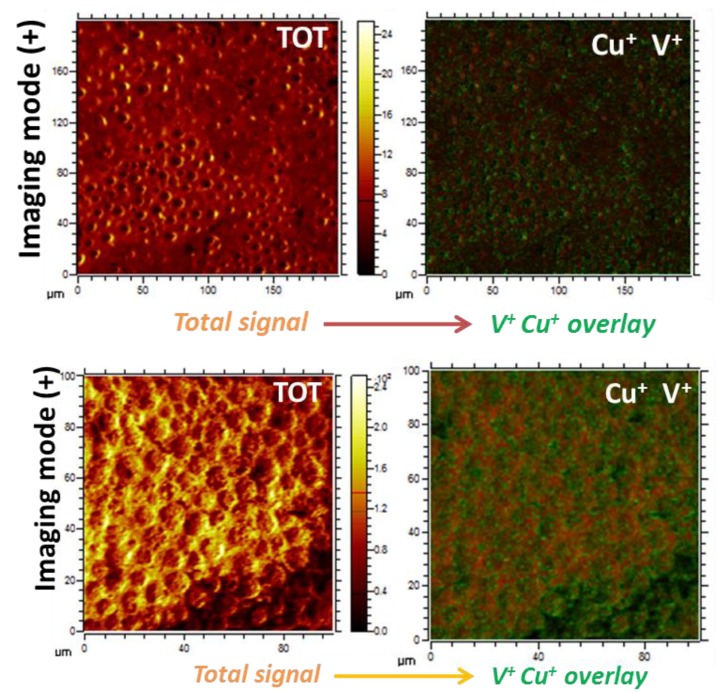
ToF-SIMS surface images obtained in imaging mode (+) are also shown and indicate the spatial distribution of the signals arising from the Cu^+^ (green) and V^+^ (red) peaks as an overlay on the right image for **BF1** (top) and **BF2** (bottom).

A comparison of the surfaces by SIMS imaging using the overlay of the signals belonging to Cu^+^ and V^+^ species and the total signal demonstrated successful immobilization of the hybrid POMs, which appeared primarily on the cavities of the film as a result of their interaction with the carboxylic groups (Figure 7).

## 3. Materials and Methods

### 3.1. Materials

Styrene (S) (99%, Sigma-Aldrich, Schnelldorf, Germany) and *t*‒butyl acrylate (tBA) (98%) (Sigma-Aldrich, Schnelldorf, Germany) were distilled under reduced pressure over calcium hydride prior to their use. Polystyrene (PS) (measured by gel permeation chromatography (GPC), weight-averaged molecular weight M_w_ = 3 × 10^6^ g mol^−1^; polydispersity index (PDI) = 1.80) was purchased from Polysciences (USA). Copper (I) bromide (CuBr) (98%), N,N,N′,N′′,N′′‒pentamethyldiethylenetriamine (PMDETA) (99%), and ethyl-2-bromoisobutyrate (EtBr) (98%) acquired from Sigma-Aldrich (Schnelldorf, Germany) and tetrahydrofuran (THF) (HPLC grade) from Scharlab (Barcelona, Spain) were used without further purification. Polymer solutions used for formation of the films were cast in round glass coverslips, 20 mm diameter, purchased from Marienfeld (Lauda-Königshofen, Germany). MilliQ-grade water was used in these studies.

### 3.2. Characterization Methods

The FTIR spectra of **1-CuV10** and **1-CuV** were recorded as KBr pellets on a Shimadzu FTIR-8400S spectrophotometer in the 400−4000 cm^−1^ spectral range. In the case of hybrid surfaces, **BF1** and **BF2** spectra were recorded using the attenuated total reflectance (ATR) mode. Diffuse reflectance UV/Vis (DR-UV/Vis) studies were performed on a UV-Vis-NIR Varian Cary 500 spectrophotometer (Varian, Palo Alto, CA, USA). Optical images of honeycomb-like samples were taken using a LEXT 3D measuring laser microscope OLS4000 Olympus in scanning XYZ mode using a MPLAPON LEXT20 lens to check the relative roughness of the samples. ^1^H-Nuclear magnetic resonance (^1^H-NMR) spectra of the synthetized copolymers were recorded at room temperature on a Bruker Avance 400 MHz spectrometer using the residual proton resonance of the deuterated solvent as internal standard. Average molar masses and molar mass distributions of the polymers were determined by size exclusion chromatography (SEC) in DMF using a Waters device equipped with two columns. Calibration was obtained using narrowly distributed polystyrene standards and DMF as the mobile phase at a flow rate of 0.5 mL min^‒1^. Scanning electron microscopy (SEM) micrographs were obtained using a Hitachi S‒4800 (150 s, 20 mA, 5.0 kV, zoom at 2000×). The samples were coated with gold, prior to scanning, using a fine coat ion sputter JFC‒1100.

Superficial characterization of POM-functionalized samples was carried out by time-of-flight secondary ion mass spectrometry (ToF-SIMS) measurements. The ToF-SIMS instrument (TOF.SIMS5) was fitted with a 30 keV Bi+ analytical beam and several sputter sources producing O_2_^+^, Ar^+^, and Cs^+^ beams, which were operated in an energy range between 0.2–2.0 KeV. Both analytical and sputtering beams were incident at 45° to the sample surface and alternated during depth profiling analyses in a dual beam mode. The sputtered secondary ions were extracted during the analytical beam pulse and introduced into the ToF analyzer by applying an extraction voltage of 2 KeV. Since the analytical pulse was very short, the ion flux was kept below the static limit (less than 1% of the surface is sputtered) to minimize surface damage during analysis. In order to neutralize any charge that might build on the surface during ion bombardment of insulating samples, a low-energy (20 eV) electron beam flooded the surface during the sputtering cycle. The analysis was performed in a small area at the center of the sputtered crater in order to avoid crater edge effects during depth profiling.

### 3.3. Synthesis of the Copolymer Polystyrene-b-poly(acrylic acid) (PS_53_-b-PAA_25_)

Synthesis of the diblock copolymer PS-b-PAA was carried out by atom transfer radical polymerization (ATRP) following previously reported procedures [37]. In brief, a polystyrene macroinitiator ([M]/[I]/[CuBr]/[L] = 50:1:1:1 where M = styrene; I = initiator, ethyl-2-bromoisobutyrate; and L = ligand, N,N,N′,N′′,N′′‒pentamethyldiethtylentriamine, PMDETA) was synthesized under N_2_ at 65 °C after degassing the reaction mixture by three freeze‒pump‒thaw cycles. The mixture was then cooled down to room temperature. The polymeric reaction was diluted in THF, and the copper salt was removed through a neutral alumina column (M_w_ = 6620 g·mol^−1^ and PDI = 1.04). Afterwards, synthesis of PS-b-PtBA was carried out ([M]/[I]/[CuBr]/[L] = 100:1:1:1 where M = *t*-butyl acrylate, I = PS‒Br, and L = PMDETA). The PS‒Br macroinitiator was previously dissolved in acetone (5 mL), and the reaction was carried out at 65 °C. M_w_ = 7757 g·mol^−1^ and PDI = 1.08. The composition of the block copolymer was determined by ^1^H-NMR to be PS_53_-b-PAA_25_. Finally, PS-b-PAA was obtained by hydrolysis of tBA groups in CH_2_Cl_2_ with trifluoroacetic acid (10 equivalents to t-butyl ester units).

### 3.4. Preparation of Breath Figure Surfaces

Polymer solutions were prepared by dissolving high-molecular-weight PS and the synthetized PS_53_-b-PAA_25_ copolymer in THF. The polymer blend employed (50 mg mL^−1^) contained 10% of PS_53_-b-PAA_25_ diblock copolymer and 90 wt% of high-molecular-weight linear PS. The films were prepared from these solutions by casting (100 µL) onto glass wafers under controlled humidity inside a closed chamber. The relative humidity (RH) was controlled by saturated aqueous solutions of KCl to obtain 80% RH. 

### 3.5. Synthesis of Polyoxometalate (POM) Precursors

[Cu(cyclam)][{Cu(cyclam)}_2_(V_10_O_28_)]·10H2O (**1-CuV10**). **1-CuV10** POM was synthesized according to a previously reported method [49]. The specific synthetic procedure was as follows: Metavanadate precursor [(CH_3_)_3_CNH_3_][VO_3_] (0.170 g, 1.00 mmol) was dissolved in aqueous 1M NaCl (20 mL), and the pH was adjusted to 4.8 with aqueous 0.5M HCl. Then, a solution of CuSO4·5H2O (0.075 g, 0.30 mmol) and cyclam (0.040 g, 0.20 mmol) in aqueous 1M NaCl (15 mL) was added dropwise. The mixture was stirred for 2 h and then filtered to remove the brown solid. The resulting dark brown solution was left to slowly evaporate in an open container at room temperature. Compound **1-CuV10** was isolated as orange block-like crystals after 6 d and identified by infrared spectroscopy. IR (cm^−1^): 3186(vs), 3165(vs), 2934(s), 2878(s), 1627(s), 1473(m), 1454(m), 1427(m), 1389(w), 1358(w), 1300(w), 1253(w), 1236(w), 1138(m), 1105(m), 1062(m), 1009(m), 960(vs), 883(m), 835(s), 748(s), 594(s), 532(s), 559(s), and 440(s).

[{Cu(cyclam)}(VO_3_)_2_]·5H_2_O (**1-CuV**). **1-CuV** POM was synthesized according to a previously reported method [47]. The specific synthetic procedure was as follows: Metavanadate precursor [(CH_3_)_3_CNH_3_][VO_3_] (0.100 g, 0.60 mmol) was dissolved in distilled water (20 mL), and the pH was adjusted to 9.0 with aqueous 1M NaOH. Then, a solution of CuSO_4_·5H_2_O (0.075 g, 0.30 mmol) and cyclam (0.040 g, 0.20 mmol) in distilled water (15 mL) was added dropwise. The mixture was refluxed for 2 h, cooled down to room temperature, and the formed dark pink precipitate was filtered. The resulting dark purple solution was left to slowly evaporate at room temperature. Compound **1-CuV** was obtained as purple prismatic crystals after 3 d and identified by infrared spectroscopy. IR (cm^−1^): 3229(s), 3165(s), 2936(m), 2878(m), 1638(m), 1474(w), 1454(w), 1442(w), 1429(w), 1389(w), 1358(w), 1312(w), 1292(w), 1253(w), 1236(w), 1105(m), 1091(w), 1074(w), 1062(w), 1016(m), 1008(m), 962(vs), 920(vs), 895(m), 883(s), 854(s), 758(s), 544(w), 521(w), 499(m), and 440(m).

### 3.6. Immobilization of Hybrid POMs onto the BFs.

Polyoxometalates were anchored onto the honeycomb patterns by immersion of the film surfaces into the corresponding solutions.

Breath Figures Surface 0 (**BF0**), reference film. A solution containing CuSO_4_.2H_2_O (0.10 mmol) and cyclam ligand (0.10 mmol) was prepared in 12 mL of deionized water. The dark purple solution was stirred for 15 min at room temperature, after which the patterned substrate was carefully immersed. After 24 h, the BF was removed from the solution, washed with deionized water repeatedly, and dried with Ar gas flow. Compared to the initial honeycomb substrate, the **BF0** sample showed a slight violet color across its surface. 

Hybrid Breath Figures Surface 1 (**BF1**). Compound **1-CuV10** was generated in situ following the synthesis described in Section 3.5. After filtering, the same procedure carried out for **BF0** was applied. The **BF1** sample presented a slight orange color.

Hybrid Breath Figures Surface 2 (**BF2**). Compound **1-CuV** was generated in situ following the synthesis described in Section 3.5. After filtering, the same procedure carried out for **BF0** was applied. The resulting **BF2** surface showed a pale purple color across its surface.

## 4. Conclusions

This work describes an effective approach to anchor polyoxovanadates functionalized by Cu(cyclam) to polymeric surfaces. The breath figure method allows formation of the honeycomb patterns in addition to obtaining carboxylic acid functionalized cavities capable of successfully anchoring the Cu(cyclam) present in both polyoxovanadates studied. This surface modification has been demonstrated by ToF-SIMS, which allows not only a surface analysis of the different species, but it also corroborates the presence of the POV in the cavities of the honeycomb polymeric surfaces.
